# Optimizing AI solutions for population health in primary care

**DOI:** 10.1038/s41746-025-01864-z

**Published:** 2025-07-12

**Authors:** Sanjay Basu, Pablo Bermudez-Canete, Tannen Christopher Hall, Pranav Rajpurkar

**Affiliations:** 1 Waymark, San Francisco, CA USA; 2 San Francisco General Hospital/University of California, San Francisco, CA USA; 3 Stanford University, Stanford, CA USA; 4 Paratus Health, Palo Alto, CA USA; 5 Harvard Medical School, Boston, MA USA

**Keywords:** Population screening, Computational models

## Abstract

Artificial intelligence (AI) has primarily enhanced individual primary care visits, yet its potential for population health management remains untapped. Effective AI should integrate longitudinal patient data, automate proactive outreach, and mitigate disparities by addressing barriers such as transportation and language. Properly deployed, AI can significantly reduce administrative burden, facilitate early intervention, and improve equity in primary care, necessitating rigorous evaluation and adaptive design to realize sustained population-level benefits.

## Introduction: the limits of AI in current primary care

Artificial intelligence (AI) has rapidly transformed healthcare, yet its application in primary care has predominantly focused on optimizing individual patient encounters. Ambient scribe systems and clinical decision-support tools aim to reduce documentation burdens and improve real-time decision-making during visits^1,2^. However, these innovations address only a fraction of primary care’s broader challenges, which include workforce shortages, fragmented care delivery, and persistent health inequities. In this viewpoint, we argue that an important next challenge for AI to benefit primary care is to address challenges of population health management. By continuously analyzing data across longitudinal patient panels, AI should be designed to enable more proactive care delivery, reduce lapses in panel management currently requiring manual tracking, and enable greater outreach during off-hours for patients who may be lost to follow-up.

## Expanding AI beyond individual patient visits

Contemporary AI applications assist clinicians during individual visits. For example, ambient scribe systems transcribe patient encounters and generate preliminary clinical notes^1^. Although these tools can improve the efficiency and quality of individual encounters, recent studies indicate that similar AI technologies should be tuned to address population-level challenges. In a recent randomized trial, an AI was designed to help primary care providers review patient registries for indication of hereditary cancer risk, manage genetic counseling and testing, and assist in follow-up—showing equivalency to current manual approaches when guiding over 3000 primary care patients^3^. These findings demonstrate that when deployed at scale, AI agents extend beyond the confines of the individual encounter to become an extension of providers into realms that involve large-scale panel management and review of patients between visits—laying the groundwork for truly proactive population-level remote monitoring.

## Population-level AI: from reactive to proactive care

A population-level AI system should continuously monitor electronic health records and other data streams that primary care providers may struggle to reconcile when under time pressure: claims data, health information exchanges, digital communications, and social service databases. Such a system should identify patients at risk for adverse outcomes even when they are not physically present at a visit or have not proactively contacted their primary care physician through an inbox message or telephone call. For instance, an AI that tracks medication fill patterns flags patients who have a chronic disease prescription refill in the electronic health record but have not picked it up according to claims data, notifying a primary care practice manager to conduct outreach and identify barriers to obtaining and adhering to the prescription^4^.

## Building provider trust in AI systems

Realizing benefits from population-level AI tools requires that the health technology industry address several key problems faced by primary care providers. Providers need to trust AI to safely reduce administrative workload and minimize missed opportunities for care. Instead of manually reviewing “no show” visit notifications, following-up with patients to reschedule visits, ensuring concerning lab results trigger appropriate medication adjustments and follow-up visits, and manually entering preventive care orders, AI tools need to demonstrate safe handling of automated messages to patients, appointment scheduling, and pending of orders, especially in cases where patients may have contraindications to standard care pathways. Nevertheless, monitoring of overall panel data—not just patients who present to in-person visits or proactively contact their provider—may help reduce errors as clinicians become overwhelmed with urgent situations and the volume of messages and alerts, and may help improve population health equity as AI tools can prioritize patients based on medical need rather than on which patients had the resources to proactively contact their provider or schedule an earlier appointment.

## Leveraging AI to improve health equity

If properly designed and supported by robust public health and primary care services, AI for population health care management has the potential to improve health equity. Traditional care models rely on patients initiating contact, a process that disadvantages individuals facing language barriers, poor access to the Internet, transportation challenges to appointments, or economic hardship, such as multiple jobs or caregiver duties that prevent time away to visit clinics. While many practices already conduct proactive outreach through text and email systems with limited engagement success, AI systems must learn from these experiences. Simply automating existing approaches will not overcome fundamental barriers to patient engagement; rather, AI must enable more personalized, culturally-appropriate, and barrier-conscious outreach strategies. AI systems capable of processing multilingual, multi-dialect text and voice messages have actively identified vulnerable patients to improve equitable outreach. For example, multilingual AI agents achieved significantly higher engagement rates among Spanish-speaking patients during colorectal cancer screening outreach compared to conventional teams^5^. Additionally, AI tools now use social service and social needs data in unique ways that may not be top-of-mind for primary care providers today. Consider a patient receiving insulin but facing food insecurity; an AI model that integrates clinical data with food support program records flags patients at high risk for hypoglycemia when food resources dwindle at month’s end, and helps care managers provide food vouchers and glucometer education during that time—when such resources are available through adequately-funded public health programs^4,6,7^.

## AI’s role in value-based care

Value-based care contracts that primary care practices have increasingly entered into further underscore the need for population-level AI. In value-based contracts, providers are financially incentivized to improve outcomes and reduce avoidable emergency department visits and hospitalizations rather than simply increase visit volume. AI systems that facilitate early intervention by flagging patients at risk for avoidable emergency department visits or hospitalizations can enable proactive prevention when properly integrated into clinical workflows. For instance, a study among Medicaid patients tested proactive ‘chase lists’ for primary care practice teams created by AI-driven care management early warning systems. The effort led to marked increases in patient care quality metrics and reductions in emergency department visits and hospitalizations among patients enrolled in the program, versus a matched control group, including a 22.9% reduction in all-cause acute events and a 48.3% reduction in ambulatory care–sensitive hospitalizations^7^. Critically, the clinical savings from the initiative assisted in the hiring of a multidisciplinary team to assist the primary care providers: an integrated approach that reduced the burden on overextended providers. While adequate funding of primary care services remains essential, AI tools can help optimize resource allocation and identify at-risk patients more efficiently within well-funded systems.

## Challenges and pitfalls in developing population-level AI

Despite its promise, developing, testing, and adopting population-level AI monitoring systems presents numerous challenges that the healthcare technology research community must address. One challenge is “regression to the mean,” wherein AI systems optimized on aggregate data have the potential to standardize care protocols so extensively that atypical high-risk cases from rare conditions are overlooked. Moreover, while algorithmic bias along race/ethnic lines is frequently discussed, AI systems risk exacerbating health inequities if not carefully designed. Depending on which patient attributes are prioritized and how outreach is organized, these tools may inadvertently favor populations already engaged with healthcare while missing those facing the greatest barriers, such as patients with inconsistent phone access or unstable housing. A less intuitive challenge is ensuring that predictive models remain adaptive. Without routine updates, static models risk becoming outdated as patient demographics and social determinants evolve. An additional fundamental challenge is data quality variability in electronic medical records. AI algorithms require accurate, consistently recorded information to function effectively, yet documentation practices vary significantly among providers. This variability in note-taking and data entry can compromise algorithm performance and potentially amplify existing disparities if certain populations receive less comprehensive documentation. Additionally, automation bias—where clinicians might over-rely on AI outputs—can reduce clinical vigilance and result in missed nuances. Transparent and interactive systems that provide clear rationales for their suggestions are essential to counter this risk.

## Ensuring rigorous evaluation and continuous improvement

Rigorous evaluation is critical. Prospective studies—such as cluster-randomized or stepped-wedge trials—are needed to assess impacts on response times, hospitalization rates, preventive care uptake, and clinician burnout, including from “alert fatigue”.

Continuous monitoring through periodic audits and user feedback can help reduce the risks for AI systems of inaccuracy, bias, harm, or lack of adaptability. In conclusion, while AI agent development for primary care providers has so far shown potential to improve individual patient encounters and provider burden, the future of primary care lies in its application to population health and overall panel management. By continuously analyzing vast amounts of data, AI can proactively identify patients with emerging risks for adverse events, triage concerns on the basis of need rather than on the basis of which patients are seen in-person or proactively send an inbox message or call the practice, and prompt timely interventions to more multidisciplinary teams to prevent adverse events. With rigorous evaluation and effective workflow integration, population-level AI promises to make primary care more efficient, equitable, and responsive to the needs of entire populations.

## Data Availability

No datasets were generated or analysed during the current study.
